# Blood-Brain Barrier Dysfunction in Small Vessel Disease Related Intracerebral Hemorrhage

**DOI:** 10.3389/fneur.2018.00926

**Published:** 2018-11-12

**Authors:** Whitney M. Freeze, Heidi I. L. Jacobs, Floris H. B. M. Schreuder, Robert J. van Oostenbrugge, Walter H. Backes, Frans R. Verhey, Catharina J. M. Klijn

**Affiliations:** ^1^Department of Psychiatry & Neuropsychology, Alzheimer Center Limburg, School for Mental Health & Neuroscience, Maastricht University, Maastricht, Netherlands; ^2^Department of Radiology & Nuclear Medicine, School for Mental Health & Neuroscience, Maastricht University Medical Center, Maastricht, Netherlands; ^3^Division of Nuclear Medicine & Molecular Imaging, Department of Radiology, Massachusetts General Hospital, Harvard Medical School, Boston, MA, United States; ^4^Department of Neurology, Center for Neuroscience, Donders Institute for Brain Cognition & Behaviour, Radboud University Medical Center, Nijmegen, Netherlands; ^5^Department of Neurology, Cardiovascular Research Institute Maastricht, Maastricht University Medical Center, Maastricht, Netherlands

**Keywords:** blood-brain barrier, intracerebral hemorrhage, cerebral small vessel disease, microbleeds, systematic review

## Abstract

**Background and Purpose:** Hypertensive vasculopathy and cerebral amyloid angiopathy are the two most common forms of cerebral small vessel disease. Both forms are associated with the development of primary intracerebral hemorrhage, but the pathophysiological mechanisms underlying spontaneous vessel rupture remain unknown. This work constitutes a systematic review on blood-brain barrier dysfunction in the etiology of spontaneous intracerebral hemorrhage due to cerebral small vessel disease.

**Methods:** We searched Medline (1946–2018) and Embase (1974–2018) for animal and human studies reporting on blood-brain barrier dysfunction associated with intracerebral hemorrhage or cerebral microbleeds.

**Results:** Of 26 eligible studies, 10 were animal studies and 16 were in humans. The authors found indications for blood-brain barrier dysfunction in all four animal studies addressing hypertensive vasculopathy-related intracerebral hemorrhage (*n* = 32 hypertensive animals included in all four studies combined), and in four of six studies on cerebral amyloid angiopathy-related intracerebral hemorrhage (*n* = 47). Of the studies in humans, five of six studies in patients with cerebral amyloid angiopathy-related intracerebral hemorrhage (*n* = 117) and seven out of nine studies examining intracerebral hemorrhage with mixed or unspecified underlying etiology (*n* = 489) found indications for blood-brain barrier dysfunction. One *post-mortem* study in hypertensive vasculopathy-related intracerebral hemorrhage (*n* = 82) found no evidence for blood-brain barrier abnormalities.

**Conclusions:** Signs of blood-brain barrier dysfunction were found in 20 out of 26 studies. Blood-brain barrier integrity deserves further investigation with a view to identification of potential treatment targets for spontaneous intracerebral hemorrhage.

## Introduction

Spontaneous intracerebral hemorrhage (ICH) is the most disabling and deadliest form of stroke (1), with an overall case fatality of 40% at 1 month, and 55% at 1 year (2, 3). Outcome after ICH has seen little improvement over the past decades (2–4). An important factor that has hampered development of specific treatment and prevention strategies is the lack of knowledge on pathophysiological processes underlying spontaneous ICH. Hypertensive vasculopathy (HV) and cerebral amyloid angiopathy (CAA), two distinct types of cerebral small vessel disease (cSVD), are thought to be the most important causes of spontaneous ICH (Box 1). In HV, degenerative alterations in the vessel wall, including arteriosclerosis or atherosclerosis, lipohyalinosis, and arteriolosclerosis affect the deep penetrating small vessels in the brain (5, 7). This type of cSVD is usually associated with ICH located in deep brain structures, including the basal ganglia and thalamus, and in the posterior fossa and brainstem (8). CAA is characterized by progressive deposition of amyloid-β (Aβ) protein in the walls of small to medium sized arteries (including arterioles) of the cerebral cortex and leptomeninges (9), and is an important cause of lobar ICH (8, 6).

Box 1 **Definitions of important terminology used throughout the paper.**

**Cerebral Small Vessel Disease:** Umbrella term covering a variety of pathological processes with various etiologies affecting the small vessels (including arteries, arterioles, venules and capillaries) of the brain. Hypertensive vasculopathy and cerebral amyloid angiopathy are the most common forms.

**Hypertensive Vasculopathy:** The vascular phenotype of chronic hypertension, including small vessel atherosclerosis, lipohyaliosis or fibrinoid necrosis, and arteriolosclerosis (5), primarily affecting the deep penetrating small vessels in the brain.

**Cerebral Amyloid Angiopathy:** Deposition of amyloid-beta in the walls of the cerebrovasculature with preferential involvement of small arterioles and capillaries of the leptomeninges and cerebral cortex, and a topographical distribution favoring posterior lobar brain regions (6).

**Spontaneous Intracerebral Hemorrhage:** Nontraumatic intraparenchymal bleeding due to spontaneous rupture of cerebral vessels.

**Cerebral Microbleeds:** Radiological manifestations of small-vessel disease which presumably correspond to small perivascular hemorrhages. Their neuropathological equivalent, cerebral microhemorrhages, correspond to erythrocyte extravasation or blood-breakdown products including hematoidin or hemosiderin.

A candidate key player in the pathophysiology of ICH is the blood-brain barrier (BBB) (10, 11). The BBB is a term used to describe the unique selective transport regulation properties of the cerebral microvasculature (12). The BBB is located along the microvascular endothelium and consists of endothelial cells connected by tight junctions, which restrict paracellular transport. Neighboring cell types include astrocytes that ensheath the microvascular wall with their endfeet, and pericytes that are embedded within the connective endothelial basement membrane. These cells, as part of the neurovascular unit network that regulates local cerebral blood flow, support and interact with the endothelial cells and are essential for the induction and maintenance of the BBB (13, 14). Upstream from capillaries, in arterioles, layers of smooth muscle cells provide BBB support in addition to other arteriolar constituents (15). Functions of the BBB include the control of molecular traffic to exclude toxins, maintenance of ion homeostasis, and control of immune surveillance and response with minimal inflammation to protect the neuronal environment (16). Previous research has led to the hypothesis that BBB dysfunction may trigger HV-type cSVD (17), and accumulating evidence also suggests a pivotal role of BBB dysfunction in CAA-related vessel pathology (18).

The objective of this systematic review is to summarize and evaluate the currently available evidence from human and animal studies to substantiate a potential role of BBB dysfunction in the underlying vasculopathy of ICH. Cerebral microbleeds (CMBs), as radiological imaging markers of cSVD and independent predictors of future ICH (19, 20), may bear a pathophysiological mechanism comparable to ICH (21). Therefore, we also included studies that investigated BBB integrity in relation to CMBs and their histological equivalent, cerebral microhemorrhages (CMHs) (22).

## Materials and methods

### Literature search strategy

Studies were identified via a systematic search of MEDLINE and EMBASE (MEDLINE 1946-March 8 2018, EMBASE 1974-March 8 2018). The terms “intracerebral hemorrhage,” “cerebral microbleed,” or “cerebral microhemorrhage” were entered in combination with the term “blood-brain barrier” (Appendix S1). Conference reports, case reports, reviews, and articles in languages other than English were not included.

### Inclusion/exclusion criteria

We included both human and animal studies investigating (1) spontaneous ICH or CMBs/CMHs and (2) BBB dysfunction preceding the hematoma in time or occurring spatially distant to the hematoma. Exclusion criteria were artificially induced hemorrhage (e.g., in experimental animal models by injection of hemorrhage mimicking or inducing compounds), traumatic ICH, hemorrhagic transformation of infarction, and secondary ICH (due to rupture of an arteriovenous malformation, dural arteriovenous fistula, cavernoma, tumor and others). We also excluded reports that investigated perihematomal BBB leakage or plasma markers of BBB dysfunction in the acute phase after ICH.

### Selection of studies and data extraction

One author (W.M.F.) performed title and abstract screening to identify studies that were eligible for inclusion, and subsequently reviewed full-text versions of these studies. In case of uncertainty with regard to abstract and full-text selection, studies were discussed with a second author (C.J.M.K.). The reference lists of the included articles were screened but this did not lead to the inclusion of additional papers. Data was extracted from all included studies by one author (W.M.F.) and independently by two other authors (F.H.B.M.S. and H.I.L.J.), who each extracted information from half of the included studies, randomly distributed. Discrepancies were resolved in consensus with the three authors (W.M.F., F.H.B.M.S., H.I.L.J.). We extracted the following data: (1) study type: animal/human, *in vivo/post-mortem*, cross-sectional/longitudinal and retrospective/prospective design; (2) hemorrhage type: ICH/CMB/CMH and underlying etiology: HV/CAA; (3) main research methods: imaging vs. biochemical and techniques used to assess BBB dysfunction and hemorrhage occurrence; (4) markers used to define BBB dysfunction and hemorrhage, if possible including hemorrhage location; (5) studied sample (percentage with hemorrhage) and control group (size, age, and sex); (6) main outcomes (numeric where possible). To quantify the strength of group differences (when possible), the standardized mean difference (Hedges' G) was computed for human observational studies that quantified and reported a mean and SD of the BBB disruption marker for the studied sample and control group. We followed the PRISMA guideline (Preferred Reporting Items for Systematic Reviews and Meta-Analyses).

### Study quality assessment

We assessed the methodological quality of the individual human case-control studies according to the Newcastle-Ottawa Scale (NOS) (23), and the quality of the studies with a case-series design with an adapted version of the NOS (Table S1) (24). Methodological quality of the individual animal studies was rated using an adapted version of the CAMARADES scale (Table S2) (25).

## Results

**Figure 1:**
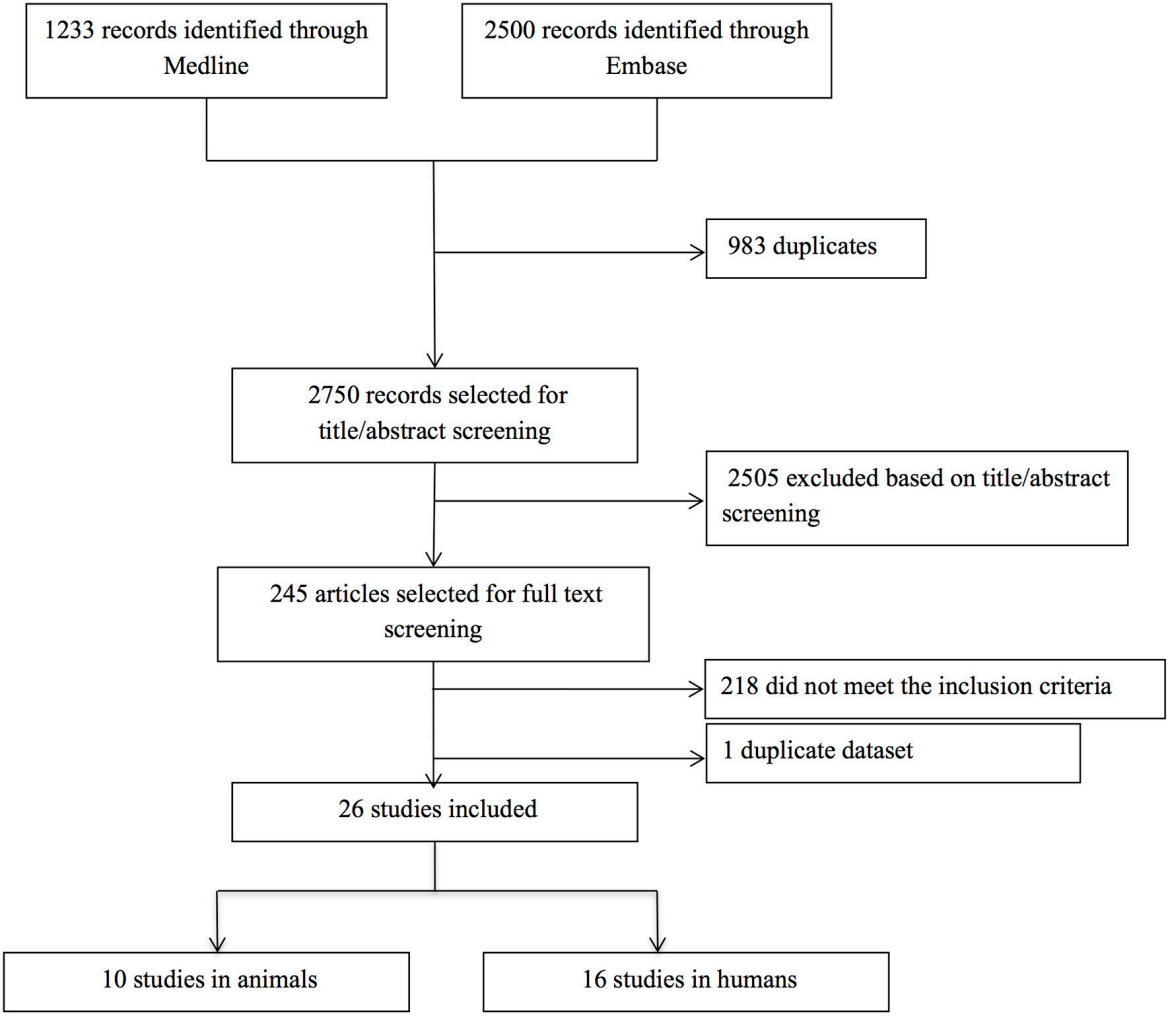
Flow chart of study selection. After title and abstract examination of 2,750 publications, 2,505 papers were excluded, leaving 245 papers to be read. Of these, 218 papers did not meet the inclusion criteria [hemorrhage was induced artificially in animals (*n* = 144) or caused by factors other than cSVD (*n* = 17), BBB dysfunction was only assessed in the peri-hematomal region (*n* = 33), or a direct measure for BBB leakage was absent (*n* = 20)], and four papers were excluded because we could not translate them (one Polish and three Chinese). One duplicate dataset was discovered, so we only included the first publication. The remaining 26 papers were deemed eligible for inclusion. No additional papers were included after screening the reference lists of the included articles.

Our search yielded a total of 2,750 publications, of which 26 studies were relevant. Ten studies described a total of 79 animals in which cSVD-related hemorrhage was modeled, and 54 animals serving as controls. Sixteen studies described 688 human patients with ICH and CMBs, and 1,080 controls without ICH and CMBs (Figure 1). The methodological quality of all studies was assessed and varied from poor (2/9) to good (9/9) (Tables S1, S2).

### Animal studies

Of the ten studies in animals, four assessed BBB dysfunction in HV-related ICH (*n* = 32), and six assessed CAA-related ICH (*n* = 47) (Tables 1, S3) (26–35).

**Table 1:** Overview animal studies.

**Study**	**cSVD type**	**Hemorrhage type**	**Study design**	**Study sample (*n*)**	**Marker of BBB dysfunction**	**Main outcome**	**Evidence for BBB dysfunction**
Lee et al. (26)	HV	ICH	L, P, *IV*	SP-SHR (7)	Leakage of contrast agent	Vascular permeability was increased in 42% of rats with ICH before ICH incidence	Yes
Bergerat et al. (27)	HV	ICH	CS, P, *PM*	Transgenic (hCETP) and non-transgenic stroke-prone Dahl-S rats (11)	Proteomic changes	Proteomic changes specific to increased stroke susceptibility profile are associated with BBB dysfunction	Yes
Jalal et al. (28)	HV	CMB	CS, P, *PM*	SP-SHR with UCAO and on a Japanese diet (5)	Plasma protein extravasation and altered gelatinase expression	IgG leakage and increased MMP-9 expression co-localized with CMBs	Yes
Schreiber et al. (29)	HV	CMB	CS, P, *PM*	SP-SHR (9)	Plasma protein deposition in vessel wall	CMBs occur for the first time several weeks after IgG vessel wall deposition can be detected	Yes
Elfenbein et al. (30)	CAA	CMB	CS, P, *PM*	Aged squirrel monkeys (4)	Plasma protein extravasation	Fibrinoid extravasation is apparent in animals with severe CAA and CMBs	Yes
Kumar-Singh et al. (31)	CAA	CMB	CS, P, *PM*	APP-sw transgenic mice (10) and PSAPP transgenic mice (*n* = 6)	Plasma protein extravasation	Transgenic animals presented with more CMBs and with more albumin and IgG leakage	Yes
Lee et al. (32)	CAA	CMB	CS, P, *PM*	Transgenic APP-sw mice (4)	Altered gelatinase expression	MMP-9 expression was found in 30% of amyloid-laden vessels and in 79% of vessels with CMBs	Yes
Lifshitz et al. (33)	CAA	CMB	CS, P, *IV*	Transgenic TGF-β1 mice (5)	Leakage of contrast agent	12% increase in transgenic mice with CMBs compared with wild type mice	Yes
Klohs et al. (34)	CAA	CMB	L*, P, IV*	Transgenic arcAβ mice (15)	Leakage of contrast agent	No difference between animals with and without CMBs	No
Winkler et al. (35)	CAA	ICH+CMB	CS, P, *PM*	Transgenic APP-23 mice (3)	HRP/Trypan blue leakage in cerebral tissue	No obvious leakage unless acute bleeding was present	No

*HV, hypertensive vasculopathy; CAA, cerebral amyloid angiopathy; ICH, intracerebral hemorrhage; CMB, cerebral microbleed; L, longitudinal; CS, cross-sectional; P, prospective; R, retrospective; n/a, not answerable; PM, post-mortem; IV, in vivo; MMP, matrix metalloproteinase; SP-SHR, stroke-prone spontaneously hypertensive rats; IgG, immunoglobulin G; UCAO, unilateral carotid artery occlusion; Dahl-S, Dahl salt-sensitive; APP, amyloid precursor protein*.

All four studies of HV-related hemorrhage found evidence for BBB dysfunction (26–29). One of these demonstrated increased vascular permeability to a gadolinium-based contrast agent on MRI that preceded ICH in time in three out of seven (42%) stroke-prone spontaneously hypertensive rats (SP-SHR) that developed ICH (26). The location of BBB leakage corresponded to the site of the ICH. The second study assessed proteomic changes in microvessels of transgenic (hCETP) hyperlipidemic Dahl salt sensitive hypertensive rats and their non-transgenic normolipidemic littermates (27). Based on age, sex and transgenic status the rats were divided into being stroke-prone (*n* = 11) and non-stroke prone (*n* = 15). Stroke susceptibility in the pre-stroke stage was associated with an increase in twelve proteins, of which nine were related to BBB integrity. A third study demonstrated CMHs and bilateral increased extravasation of the plasma protein immunoglobulin G (IgG) with co-localized matrix metalloproteinase-9 (MMP-9) immunoreactivity in the external capsule, corpus callosum, and internal capsule in SP-SHR with unilateral carotid artery occlusion and on a stroke-permissive diet (Japanese-style) (*n* = 5) compared with sham-operated SP-SHR on a regular diet (*n* = 5) (28). The fourth study identified deposition of IgG in vessel walls within the cortex, basal ganglia, hippocampus and corpus callosum of SP-SHR (*n* = 9) that preceded the formation of CMHs (29).

Four of six studies examining animal models of CAA found evidence for BBB dysfunction (30–35). One study identified fibrinoid extravasation and CMHs in some CAA affected vessels in the forebrain cortex of old squirrel monkeys with CAA (*n* = 4), but not in a young squirrel monkey with no CAA (*n* = 1) (30). A second study demonstrated BBB leakage in the form of increased albumin and IgG plasma protein extravasation in TG2576 (APP/Sw) (*n* = 10) and bigenic PSAPP (APP/Sw X PS1_M146L_) (*n* = 6) mice with increased CMH load compared with nontransgenic controls (*n* = 6) (31). The third study showed MMP-9 immunoreactivity in 30% of CAA affected vessels and in 79% of vessels with CMHs in old Tg2576 mice (*n* = 4) while no MMP-9 immunoreactivity could be detected in young Tg2576 (*n* = 2) or old wild type mice (*n* = 2) (32). The fourth study found evidence for BBB dysfunction in heterozygous TGF-β mice (*n* = 5) with CMHs on histopathology in the form of a 12% increase in BBB permeability to a gadolinium-based contrast agent compared with wild type littermates (*n* = 5) (33). In contrast, another MRI study did not find any difference in BBB leakage of gadolinium-based contrast material in transgenic arcAβ mice with higher CMB frequencies (*n* = 15) compared with wild type controls (*n* = 15) (34). The sixth study found no evidence for leakage of horseradish peroxidase or Trypan blue dye at sites beyond the vicinity of the hemorrhage in transgenic APP23 mice (*n* = 3) and wild type littermates (*n* = 3) (35).

### Human studies

Of the sixteen included studies, one assessed BBB dysfunction in HV-related ICH (*n* = 82) (36) six in CAA-related ICH (*n* = 117) (37–42), and nine did not specify the underlying etiology of the ICH (*n* = 489; Tables 2A, 2B, S4) (44–52).

**Table 2A:** Overview human studies.

**Study**	**cSVD type**	**Determination cSVD type**	**Hemorrhage type**	**Study design**	**Study sample (n)**	**Marker of BBB dysfunction**	**Main outcome**	**Evidence for BBB dysfunction**
Plesea et al. (36)	HV	Hypertensive patients	ICH	CS, n/a, *PM*	ICH patients (82)	Integrity of vascular endothelium	The endothelial structures of arteries, arterioles and capillaries were intact	No
Hernandez-Guillamon et al. (38)	CAA	Boston criteria (43)	ICH	CS, P, *PM, IV*	CAA with ICH (6 + 33)	Altered gelatinase expression	MMP-2, but not MMP-9, was increased in and around CAA-affected vessels in ICH patients. No differences were found in plasma or in cerebral tissue	Yes
Zhao et al. (42)	CAA	*PM* pathologic examination	ICH+CMB	CS, P, *PM*	CAA with/without ICH (*n* = 6), CAA with AD (*n* = 4)	Altered gelatinase expression	The percentage of vessels with MMP-9 and MMP-9 expression were increased in CAA-affected vessels	Yes
Duits et al. (41)	CAA	Patients had lobar CMBs	CMB	CS, P, *IV*	AD/VaD with CMBs^*^ (34)	Altered gelatinase expression	MMP-9 levels were decreased in CSF of individuals with AD with CMBs. TIMP-1 and TIMP-2 were similar to cognitively normal controls	Yes
Cheng et al. (40)	CAA	*PM* pathologic examination	CMB	CS, P, *PM*	CAA with CMBs (9)	Tight junction protein expression	Occludin expression was reduced in leptomeningeal and cerebral vessels	Yes
Hartz et al. (37)	CAA	Boston criteria (43)	ICH+CMB	CS, P, *IV*	CAA with ICH or CMBs (19)	Leakage of contrast agent	Contrast agent leakage was apparent in 11% of ICH cases	Yes
van Assema et al. (39)	CAA	Patients had lobar CMBs	CMB	CS, P, *IV*	AD with lobar CMBs (6)	Altered pgp expression	Pgp expression was not different between AD patients with and without CMBs	No

* *Study samples partly overlap for these studies*.

*cSVD, cerebral small vessel disease; BBB, blood-brain barrier; HV, hypertensive vasculopathy; CAA, cerebral amyloid angiopathy; ICH, intracerebral hemorrhage; CMB, cerebral microbleed; L, longitudinal; CS, cross-sectional; P, prospective; R, retrospective; n/a, not answerable; PM, post-mortem; IV, in vivo; MMP, matrix metalloproteinase; AD, Alzheimer disease; VaD, vascular dementia; TIMP, tissue inhibitor of metalloproteinase; CSF, cerebrospinal fluid*.

**Table 2B:** Overview human studies (continued from Table 2A).

**Study**	**cSVD type**	**Determination cSVD type**	**Hemorrhage type**	**Study design**	**Study sample (n)**	**Marker of BBB dysfunction**	**Main outcome**	**Evidence for BBB dysfunction**
Aksoy et al. (44)	40% HV, 20% CAA, 40% other	n/a	ICH	CS, P, *IV*	ICH patients (25)	Leakage of contrast agent	Contrast agent leakage was apparent in contralateral hemisphere in 48% of ICH cases	Yes
Kidwell et al. (45)	Unspecified, primary ICH	n/a	ICH	CS, R, *IV*	ICH patients (46)	Leakage of contrast agent	Contrast agent leakage was apparent in CSF in 85% of ICH cases	Yes
McCourt et al. (46)	Unspecified, primary ICH	n/a	ICH	CS, P*, IV*	ICH patients (53)	Leakage of contrast agent	Contrast agent leakage was increased in ipsilateral hemisphere compared with contralateral hemisphere	Yes
Xu et al. (47)	Unspecified, primary ICH	n/a	ICH	CS, n/a, *IV*	ICH patients (54)	Leakage of contrast agent	Contrast agent leakage did not differ in ipsilateral hemisphere compared with contralateral hemisphere	No
Zhang et al. (48)	Unspecified	n/a	CMB	CS, P, *IV*	AD with CMBs (47)	Serum VEGF levels	Serum VEGF levels were increased in AD patients with CMBs	Yes
Shams et al. (49)	Unspecified	n/a	CMB	CS, P, *IV*	Memory clinic patients with CMBs (214, of which 118 with multiple CMBs)	Altered CSF/serum albumin ratio	Albumin ratio was increased in memory clinic patients with multiple CMBs	Yes
Poliakova et al. (50)	Unspecified	n/a	CMB	CS, P, *IV*	Patients with cognitive decline with CMBs (15)	Altered CSF/serum albumin ratio	Albumin ratio was increased in patients with cognitive decline with CMBs	Yes
Tran et al. (51)	Unspecified	n/a	ICH+CMB	CS, R, *PM*	ICH patients (9)	Tight junction protein expression	Claudin-1 expression was decreased in cerebral vessels in ICH cases	Yes
Goos et al. (52)	Unspecified	n/a	CMB	CS, P*, IV*	AD with CMBs^*^ (26)	Altered CSF/serum albumin ratio	Albumin ratio was not different between AD patients with and without CMBs	No

* *Study samples partly overlap for these studies*.

*cSVD, cerebral small vessel disease; BBB, blood-brain barrier; HV, hypertensive vasculopathy; CAA, cerebral amyloid angiopathy; ICH, intracerebral hemorrhage; CMB, cerebral microbleed; L, longitudinal; CS, cross-sectional; P, prospective; R, retrospective; n/a, not answerable; PM, post-mortem; IV, in vivo; AD, Alzheimer disease; CSF, cerebrospinal fluid; VEGF, vascular endothelial growth factor*.

One study examined HV-related ICH patients *post-mortem* (*n* = 82) and reported endothelial integrity of cerebral arteries, arterioles and capillaries, and as such found no evidence for BBB dysfunction (36).

Of six studies that assessed CAA-related BBB dysfunction, five studies found evidence for ICH-associated BBB dysfunction. Three studies assessed BBB abnormalities as defined by altered gelatinase levels in cortical vessels (38, 42), cerebral tissue (38), plasma (38), and CSF (41), in patients with CAA. The first study identified an increased percentage of MMP-2 positive cortical vessels in moderately to severely Aβ affected vessels from CAA patients compared with not to mildly affected cortical vessels of the same patients (*n* = 6) and cortical vessels of non-CAA controls (*n* = 3). No differences were found for MMP-9. In the same study, MMP-2 and MMP-9 plasma concentrations were measured *in vivo* but their levels did not differ between CAA-related ICH (*n* = 33) and non-CAA controls (*n* = 21). Besides, *post-mortem* tissue pro-MMP-2 and pro-MMP-9 protein content was not different between individuals with CAA-related ICH (*n* = 4) and non-CAA controls (*n* = 3) (38). A second study found that 79% of CAA-affected vessels were immunopositive for MMP-9 in CAA cases with (*n* = 4) and without (*n* = 6) Alzheimer disease (AD), while only 5% of control vessels were immunopositive for MMP-9 (*n* = 4 non-CAA control cases). Moreover, MMP-9 immunoreactivity in the vessel wall was significantly increased in CAA vessels at late stages (stages 3 and 4) compared with early stages (stages 0–1), but no MMP-9 differences were observed between non-affected vessels of CAA patients and non-CAA controls (42). A third study found lower MMP-9 concentrations in CSF of individuals with AD or vascular dementia (VaD) with lobar CMBs (*n* = 32) compared with AD or VaD patients without lobar CMBs (*n* = 27) and cognitively normal controls (*n* = 26). Tissue inhibitors of matrix metalloproteinase (TIMP-2) CSF levels were elevated in AD/VaD without CMBs (*n* = 27) compared with cognitively normal controls (*n* = 26) and AD/VaD patients with CMBs (*n* = 34), but no differences were found for TIMP-1 (41).

Two other studies examining CAA patients found evidence for BBB dysfunction. The first study in the form of decreased tight junction protein (TJP) occludin immunoreactivity in capillaries from the frontal cortex, and reduced levels of TJPs occludin and ZO-1 in leptomeningeal vessels as measured with Western blot analyses in patients with CAA (*n* = 9) compared with non-CAA controls (*n* = 10) (40). The second study used MRI to identify gadolinium-based contrast agent leakage through the BBB on T1-weighted images in a subset (*n* = 2 out of 19, 11%) of individuals with CAA (37). In contrast, one study in patients with Alzheimer disease with (*n* = 6) and without (*n* = 12) lobar CMBs found no difference in p-glycoprotein expression (39).

Seven out of nine studies found evidence for BBB dysfunction without distinguishing between HV- and CAA-related ICH. Two of these found gadolinium-based contrast agent leakage on MRI in 48% to 85% of patients (44, 45). The third and fourth study used an iodine-based contrast agent to measure BBB dysfunction with computed tomography. One of these studies found increased permeability in the hemisphere ipsilateral to the ICH relative to the contralateral hemisphere (*n* = 53) within 26 h after symptom onset (46). In contrast, the other study could not identify any difference in BBB permeability between the hemisphere ipsilateral to the ICH and the contralateral hemisphere (*n* = 54) between 24 and 72 h after ICH onset (47). No control group was included in any of these imaging studies. The fifth study described decreased immunoreactivity of TJP claudin-1 on visually inspected sections of basal ganglia tissue of patients with ICH (*n* = 9) compared to sections of non-ICH controls (*n* = 10) (51). The sixth study found increased CSF/serum albumin ratios in memory clinic patients with multiple CMBs (*n* = 118), but not in memory clinic patients with single CMBs (*n* = 96) compared to those without CMBs (*n* = 825) (49). The seventh study demonstrated increased vascular endothelial growth factor levels in serum of AD patients with CMBs (*n* = 47) vs. AD patients without CMBs (*n* = 99) (48). The eighth study showed increased BBB dysfunction in the form of an increased CSF/serum albumin ratio in patients with cognitive decline with CMBs (*n* = 15) compared with patients with cognitive decline but no CMBs (*n* = 13) (50). In contrast, the last study could not identify any difference in BBB dysfunction measured as CSF/serum albumin ratios between AD patients with CMBs (*n* = 26) and AD patients without CMBs (*n* = 26) (52).

The effect size of the group difference between the studied sample and control sample of 5 human studies that quantified the mean (SD) of markers of BBB dysfunction is visualized in Figure S1. Three of the five studies report no difference in BBB dysfunction between patients and controls in these quantified measurements, which is in line with the results described in the previous paragraphs (38, 39, 52). However, one of these studies did find a difference between patients and controls when using another measurement method which we could not include in our figure (percentage of MMP-2 positivity within and around vessels in moderately to severely Aβ affected vessels from ICH patients) (38). Because of the large heterogeneity of markers used to assess BBB dysfunction and the differences in control samples that were used, we did not proceed to meta-analysis.

## Discussion

In this systematic review we found support for a possible role of BBB dysfunction in the pathophysiology of cSVD-related ICH and CMBs/CMHs in 20 out of 26 studies. We found evidence supportive of a role for BBB dysfunction in both animal and human studies, and in HV-related ICH or CMBs/CMHs and CAA-related ICH or CMBs/CMHs. Signs of BBB disruption were found in studies with *in vivo* BBB assessment, including MRI, CT and CSF/serum analysis, as well as in studies using *post-mortem* histopathological or proteomic analyses to assess BBB dysfunction. Based on the available observations in these studies, we postulate that multiple pathways may contribute to BBB dysfunction, which may in part be caused by the underlying pathology. Degradation of the basement membrane, loss of tight junction proteins, and plasma protein deposition within the vessel wall probably contribute to vessel wall instability. These vessel changes may interfere with the vessels' autoregulatory ability to respond to changes in blood pressure, which may in turn predispose to rupture in the context of hypertension or CAA. Interestingly, animal studies suggest that BBB dysfunction precedes ICH or CMB/CMH occurrence, but it remains unclear whether this relationship is causal.

The studies included in this review used a wide variety of methods to, presumably, measure BBB dysfunction. Although we reported differences in study quality according to standard assessment scales, we cannot compare the sensitivity and specificity of each individual BBB assessment approach. As a result, and because of the wide variety of the included animal models and types of patients, it is difficult to extrapolate the findings in this review outside the context of each individual study. This heterogeneity is also reflected by the variation in effect sizes of differences between cases and controls that we computed for five human studies (Figure S1). Some markers may be more strongly associated with hemorrhagic brain pathology than others, which may explain the relatively large effect size of the study by Zhang et al. (48). In addition, the validity of the markers that were identified by the studies as measuring BBB dysfunction remains unclear. For example, multiple studies measured the CSF-albumin ratio as a proxy for BBB dysfunction, but previous research has suggested that this marker is more strongly associated with blood-CSF-barrier dysfunction at the level of the choroid plexus rather than with BBB dysfunction (53–56).

Although the majority of studies (20 out of 26) found support for the possibility that blood-brain barrier dysfunction may play a role in cSVD-related intracerebral hemorrhage, there were six studies that did not find this association. Two of the four human studies that did not find indications for BBB dysfunction compared AD patients with CMBs to AD patients without CMBs (39, 52). A possible source of bias in these studies is latent presence of HV and CAA in AD without CMBs, such that the control AD group also has BBB dysfunction to some degree. Methodological issues, such as low sensitivity of the marker of BBB dysfunction and statistically underpowered analyses due to restricted sample size may also have played a role.

Animal studies are considered a valuable tool to study the temporal relationship of pathophysiological processes. However, the majority of animal studies included in this review did not have a longitudinal but a cross-sectional design. Most of the cross-sectional studies found BBB abnormalities in concurrence with the presence of CMHs (28, 30–33, 35), but some observed BBB changes in animal models of HV or CAA before they reached the age at which hemorrhages typically occur (27, 29). Only two studies assessed the relation between BBB dysfunction and occurrence of CMBs or ICH longitudinally. One study did not identify BBB alterations in relation to CMBs (34), while the other study was able to monitor ICH development and preceding co-localized BBB dysfunction *in vivo* (26). A recent case-report of a patient with probable CAA showed development of a new CMB exactly at the location of gadolinium leakage on 7T MRI (11). These findings, although the evidence is limited, suggest that BBB dysfunction may precede cerebral hemorrhage.

Strengths of this review include the systematic literature search and inclusion of both human and animal studies, which allowed us to summarize the evidence of BBB dysfunction assessed by a variety of methods and in multiple and variable cohorts of cSVD related ICH patients and animals. In addition, the inclusion of animal studies allowed interpretations in terms of disease mechanisms and could provide a first indication of the temporal relationship between BBB dysfunction and cerebral hemorrhage.

Our systematic review also has limitations. The title and abstract screening were performed by a single author (W.M.F.) which may have resulted in some form of bias with regard to the study selection. However, in case of uncertainty, the abstract or text was discussed with a second author (C.J.M.K.). Many of the included studies had a relatively small sample size, eight studies lacked a control group, and results were not quantified in five studies. Some of the CMBs rated on MRI, especially beyond the context of CAA, may correspond to other lesion types, such as microinfarcts with a hemorrhagic component, which may bias the results (57). Furthermore, caution is warranted since negative findings in general and presence of hemorrhage in cSVD animal models are likely to be underreported, leading to publication bias and selective reporting within studies. Moreover, results on various transgenic animal models with CAA were reported but these models may not be specific to cSVD since the animals also exhibit AD pathology, and several studies examined animals at a relatively young age. Although the findings in animal models are valuable for understanding disease physiology, these models do not perfectly mimic human disease. While the human studies suggest possible involvement of BBB dysfunction in ICH or CMB formation, their cross-sectional nature hampers the ability of making any causal inferences. We only identified one human study that assessed HV-related ICH, so future studies on this topic are warranted. Finally, although previous work has suggested that CMBs are predictors of future ICH (19), it is not yet clear whether these two types of hemorrhage constitute similar pathophysiological foundations (58).

To increase insights into the role of BBB dysfunction in the pathophysiology of ICH and CMBs/CMHs, dedicated prospective studies of animal models of CAA and HV with serial *in vivo* neuroimaging examinations combined with *post-mortem* BBB assessment are needed. This should be combined with longitudinal *in vivo* monitoring of BBB integrity in various cohorts and *post-mortem* assessments in humans. Such studies are, unfortunately, hard to establish because it will be difficult to achieve a reasonable sample size. Besides the role of BBB dysfunction in vessel rupture, its involvement in other cSVD-associated brain injury types and cognitive decline should be considered as well. Previous studies have already linked BBB dysfunction to brain atrophy, lacunar infarcts, WMH pathogenesis and cognitive impairment (59–62), emphasizing the possible clinical relevance of BBB dysfunction beyond ICH pathogenesis. The development of more sensitive methods to measure and localize BBB dysfunction *in vivo* may identify individuals at risk for ICH and other types of cSVD-related brain injury. Importantly, supporting BBB integrity may be a potential target in developing strategies to prevent ICH and CMBs/CMHs in the context of cSVD, and monitoring BBB dysfunction could be a method to evaluate the efficiency of treatment strategies.

## Conclusion

This systematic review suggests that BBB dysfunction plays a role in the pathophysiology of cSVD-related ICH and CMBs/CMHs. The hypothesis that BBB dysfunction precedes ICH or CMB/CMH formation needs confirmation in larger longitudinal studies in humans.

## Author contributions

WF, RvO, and CK conceived and designed the study. WF, HJ, and FS acquired the data. WF, HJ, FS, RvO, WB, FV, and CK interpreted the results. WF drafted the manuscript. HJ, FS, RvO, WB, FV, and CK made critical revisions.

### Conflict of interest statement

The authors declare that the research was conducted in the absence of any commercial or financial relationships that could be construed as a potential conflict of interest.
