# Supplementary figures and images for: Blood-Brain Barrier Dysfunction in Small Vessel Disease Related Intracerebral Hemorrhage
Source: Front Neurol. 2018 Nov 12;9:926. doi: 10.3389/fneur.2018.00926 (PMC6240684; doi:10.3389/fneur.2018.00926)

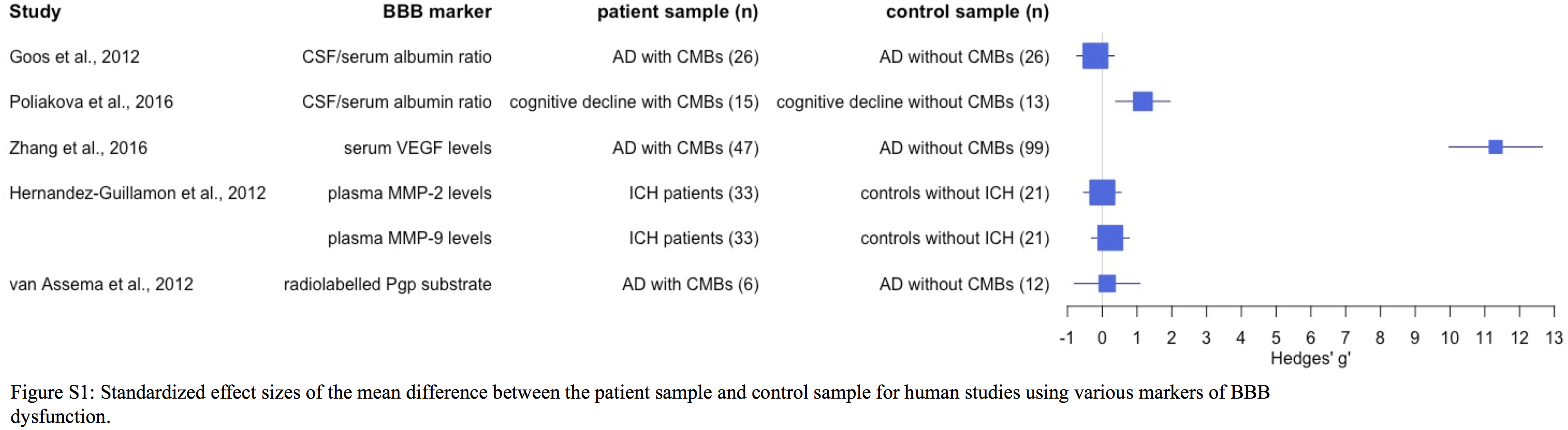

Supplement: Supplementary file 1 [file Image_1.TIFF]
