# Supplementary material for: Blood-Brain Barrier Dysfunction in Small Vessel Disease Related Intracerebral Hemorrhage
Source: Front Neurol. 2018 Nov 12;9:926. doi: 10.3389/fneur.2018.00926 (PMC6240684; doi:10.3389/fneur.2018.00926)
Supplement: Supplementary file 2 [file Table_1.DOCX]

**Appendix S1**

**Search strategy implemented in OVID MEDLINE and EMBASE**

1. exp blood-brain barrier/
2. Blood-brain barrier.tw.
3. exp capillary permeability/and exp brain/
4. exp microcirculation/and exp brain/
5. exp endothelium, vascular/and exp brain/
6. exp tight junction/and exp brain/
7. (capillary permeability adj10 (cerebral or brain)).tw.
8. (vascular permeability adj10 (cerebral or brain)).tw
9. (microcirculation adj10 (cerebral or brain)).tw.
10. (vascular endothelium adj10 (cerebral or brain)).tw
11. (tight junction adj10 (cerebral or brain)).tw.
12. (blood adj5 vessel adj5 wall).tw.
13. BBB.tw
14. or/1-13
15. exp intracerebral h?emorrhage/
16. (h?emorrhag$ adj5 (cerebral or brain or intracerebral or intraparenchymal or intracranial)).tw.
17. (h?ematoma adj5 (cerebral or brain or intracerebral or intraparenchymal or intracranial)).tw.
18. (bleeding adj5 (cerebral or brain or intracerebral or intraparenchymal or intracranial)).tw.
19. (microbleed$ adj5 (cerebral or brain or intracerebral or intraparenchymal or intracranial)).tw.
20. (microh?emorrhag$ adj5 (cerebral or brain or intracerebral or intraparenchymal or intracranial)).tw.
21. microbleed$.tw.
22. microh?emorrhag$.tw.
23. or/15-22
24. 14 and 23
25. limit 24 to review
26. 24 not 25

Table S1: Quality assessment Human Studies according to Newcastle-Ottawa scale (Herzog et al.; Wells)

|  |  | **Selection** |  |  |  | **Comparability** | | **Outcome/Exposure** | | |  |  |
| --- | --- | --- | --- | --- | --- | --- | --- | --- | --- | --- | --- | --- |
|  | Study type | Representativeness of the sample | Sample size | Non-respondents | Ascertainment of exposure | Study controls for (most important factor) | Study controls for (additional factor) | Assessment of outcome | | Statistical test | | Score total |
| **Plesea et al., 2005** | Case-series | 0 | 0 | n/a | 2 | 0 | 0 | 0 | | 0 | | 2/9 |
| **Hartz et al., 2012** | Case-series | 1 (consecutive series of cases) | 0 | 0 | 2 | 0 | 0 | 2 | | 1 | | 6/10 |
| **Aksoy et al., 2013** | Case-series | 0 | 0 | 0 | 2 | 0 | 0 | 2 | | 1 | | 5/10 |
| **Kidwell et al., 2011** | Case-series | 1 (consecutive series of cases) | 1 (explorative study) | 0 | 2 | 1 (MRI timing) | 1 (contrast dosage) | 2 | | 1 | | 9/10 |
| **McCourt et al., 2015** | Case-series | 1 (random sampling) | 1 (n>50) | 0 | 2 | 1 (hypertension) | 0 | 2 | | 1 | | 8/10 |
| **Xu et al., 2017** | Case-series | 1 (consecutive series of cases) | 1 (n>50) | 0 | 2 | 0 | 0 | 2 | | 1 | | 7/10 |
|  | Study type | Is the case definition adequate? | Representativeness of cases | Selection of Controls | Definition of controls | Study controls for (most important factor) | Study controls for (additional factor) | Ascertainment of exposure | Same method of ascertainment for cases and controls | | Non-response rate | Score total |
| **Hernandez-Guillamon et al., 2012 part 1 (*in vivo*)** | Case-control | 1 (MRI) | 1 (consecutive series of cases) | 1 | 0 | 0 | 0 | 1 | 1 | | 1 | 6/9 |
| **Hernandez-Guillamon et al., 2012 part 2 +3 (*post-mortem*)** | Case-control | 1 (pathologic confirmation) | 0 | 1 | 0 | 0 | 0 | 1 | 1 | | 1 | 5/9 |
| **Cheng et al., 2014** | Case-control | 1 (iron stain) | 1 (random sample) | 1 | 0 | 1 (age-matched) | 1 (matched for sex, post-mortem interval, and sample preparations) | 1 | 1 | | 1 | 8/9 |
| **Duits et al., 2015** | Case-control | 1 (MRI) | 0 | 1 | 1 | 1 (age-matched) | 1 (sex-matched) | 1 | 1 | | 0 (missing data) | 7/9 |
| **Zhao et al., 2015** | Case-control | 0 (only symptomatic haemorrhages rated) | 0 | 1 | 0 | 1 (age-matched) | 0 | 1 | 1 | | 1 | 5/9 |
| **Van Assema et al., 2012** | Case-control | 1 (MRI) | 0 | 1 | 1 | 0 | 0 | 1 | 1 | | 1 | 6/9 |
| **Zhang et al., 2016** | Case-control | 1 (MRI) | 1 (consecutive series of cases) | 1 | 1 | 1 (age-adjusted analysis) | 1 (sex, cardiovascular risk factors, lacunar infarction, WMH adjusted analyses) | 1 | 1 | | 1 | 9/9 |
| **Shams et al., 2016** | Case-control | 1 (MRI) | 1 (consecutive series of cases) | 1 | 1 | 0 | 0 | 1 | 1 | | 1 | 7/9 |
| **Poliakova et al., 2016** | Case-control | 1 (MRI) | 0 | 0 | 1 | 0 | 0 | 1 | 1 | | 0 (missing data) | 4/9 |
| **Goos et al., 2012** | Case-control | 1 (MRI) | 0 | 1 | 1 | 1 (Age-matched) | 1 (sex-matched) | 1 | 1 | | 0 (missing data) | 7/9 |
| **Tran et al., 2016** | Case-control | 1 (symptoms and H&E) | 0 | 1 | 1 | 0 | 0 | 1 | 1 | | 1 | 6/9 |

Abbreviation key: n/a, not applicable; MRI, magnetic resonance imaging; WMH, white matter hyperintensities; H&E, hematoxylin and eosin.

Table S2: Quality assessment animal studies according to CAMARADES scale (Sena et al., 2007)

|  | Publication in peer-reviewed journal | Statement of control of temperature | Randomization of treatment or control | Allocation concealment | Blinded assessment of outcome | Type of anesthetics specified * | Use of animals with hypertension or CAA* | Sample-size calculation | Statement of compliance with regulatory requirements | Statement regarding possible conflict of interest | Score total |
| --- | --- | --- | --- | --- | --- | --- | --- | --- | --- | --- | --- |
| **Lee et al., 2007** | 1 | 0 | n/a | n/a | n/a | 1 (isoflurane during MRI) | 1 | 0 | 1 | 1 | 5/7 |
| **Bergerat et al., 2011** | 1 | 0 | n/a | n/a | n/a | 0 (‘general anesthesia’) | 1 | 0 | 1 | 1 | 4/7 |
| **Jalal et al., 2012** | 1 | 1 | n/a | n/a | n/a | 1 (2% isoflurane during surgery/blood collection and 50mg/kg pentobarbital before tissue collection) | 1 | 0 | 1 | 1 | 6/7 |
| **Schreiber et al., 2012** | 1 | 0 | n/a | n/a | n/a | 1( 1%-1.5% isoflurane during MRI) | 1 | 0 | 1 | 1 | 4/7 |
| **Winkler et al., 2001** | 1 | 0 | n/a | n/a | n/a | 1 (pentobarbital overdose before tissue collection) | 1 | 0 | 0 | 1 | 4/7 |
| **Elfenbein et al., 2007** | 1 | 0 | n/a | n/a | n/a | 1 (15mg/kg ketamine and 100mg/kg pentobarbital before tissue collection) | 1 | 0 | 1 | 1 | 5/7 |
| **Klohs et al., 2013** | 1 | 1 | n/a | n/a | n/a | 1 (3% isoflurane before MRI, 1.5% isoflurane during DCE-MRI) | 1 | 0 | 1 | 1 | 6/7 |
| **Kumar-Singh et al., 2005** | 1 | 0 | n/a | n/a | n/a | n/a (animals were euthanized by cervical dislocation) | 1 | 0 | 1 | 1 | 4/6 |
| **Lee et al., 2003** | 1 | 0 | n/a | n/a | n/a | 0 (not mentioned in text) | 1 | 0 | 0 | 1 | 3/7 |
| **Lifshitz et al., 2012** | 1 | 0 | n/a | n/a | n/a | 1 (1-3% isoflurane during MRI) | 1 | 0 | 1 | 1 | 5/7 |

* Item adjusted from original CAMARADES scale (Sena et al.) to better reflect study quality of non drug-trial studies in hypertension and CAA animal models

Abbreviation key: MRI, magnetic resonance imaging; DCE-MRI, dynamic contrast-enhanced magnetic resonance imaging; CAA, cerebral amyloid angiopathy; n/a, not applicable.

Table S3: Methodology in animal studies

| **Study** | **Hemorrhage marker (% study sample with hemorrhage)** | **Assessment method hemorrhage** | **Location hemorrhage** | **BBB dysfunction marker** | **Reason authors chose this marker** | **Assessment method BBB dysfunction** | **Mean(sd) marker of BBB dysfunction study sample** | **Mean(sd) marker of BBB dysfunction control sample** | **Control sample** | **Study sample** | | | **Control sample** | | |  |
| --- | --- | --- | --- | --- | --- | --- | --- | --- | --- | --- | --- | --- | --- | --- | --- | --- |
|  |  |  |  |  |  |  |  |  |  | **size** | **age** | **% male** | **size** | **age** | **% male** | |
| **Lee et al., 2007** | ICH symptoms/ MRI (70%) | T2*-weighted images | Cortex, corpus callosum, striatum | CE lesions | Contrast agent extravasates when BBB dysfunctional | DCE-MRI | 42% | n/a | Absent | 7 | n/a | 100 | n/a | n/a | n/a | |
| **Bergerat et al., 2011** | Earlier knowledge on animal model (n/a) | n/a | n/a | Expression of various proteins | Proteins have been reported in literature to be part of BBB, and their increase is expected to alter BBB function | Immunohistochemistry and QSPEC analysis | Aquaporin-4, G-protein α12, Collagen XII, Carbonic anhydrase-4, NHE-3-regulator-2, syntrophin α1, Filamin B, Transferrin, Laminin α1 | n/a | Young nontransgenic dahl-s rats | 11 | 4.5 months | 27 | 15 | 2-4.5 months | 60 | |
| **Jalal et al., 2012** | Microscope (n/a) | H&E | 100% white matter | MMP-2, MMP-3, MMP-9, IgG immunoreactivity | IgG extravasates when BBB disrupted, MMPs have been associated with BBB disruption | Immunoblotting & immunohistochemistry | n/a | n/a | Sham operated SP-SHR on a regular diet | 5 | 16-17 weeks | 100 | 5 | 16-17 weeks | 100 | |
| **Schreiber et al., 2012** | Microscope, MRI (n/a) | H&E, T2-weighted images | Basal ganglia, cortex, hippocmapus, corpus callosum | IgG immunoreactivity | IgG extravasates when BBB is disrupted | Immunohistochemistry | n/a | n/a | Absent | 9 | 18-36 weeks | 100 | n/a | n/a | n/a | |
| **Elfenbein et al., 2007** | Microscope (n/a) | DAB-enhanced Perls’ method | n/a | Eosinphilic fibrinoid | Fibrin extravasates when BBB is disrupted | H&E | n/a | n/a | Young squirrel monkey | 4 | 20 | 100 | 1 | 7 | 100 | |
| **Kumar-Singh et al., 2005** | Microscope (n/a) | H&E, Perls’ blue | n/a | Albumin and IgG extravasation | Albumin and IgG extravasate when BBB is disrupted | Immunohistochemistry | Albumin: 0.6+/-0.07 for young, 1.13+/-0.12 for adult, and 1.67+/-0.14 for old mice; IgG: 0.54+/-0.06 for young, 1.05+/-0.13 for adult, and 1.73+/-0.13 for old mice | Albumin: 0.1+/-0.08 for young, 0.35+/-0.12 for adult, and 0.8+/-0.16 for old mice;  IgG: 0.05+/-0.04 for young, 0.3+/-0.11 for adult, and 0.5+/-0.12 for old mice | Nontransgenic mice | 16 | 14 months | 44% | 6 | 18 months | n/a | |
| **Lee et al., 2003** | Microscope (n/a) | Perls’ blue | n/a | Vessels /CMBs positive for MMP-9 immunoreactivity | MMPs have been associated with BBB disruption | Immunofluorescence | 30% of CAA affected vessels and 79% of CMBs | 0% MMP-9 immunoreactivity | Wild type mice + young APPsw mice | 4 | 15-18 months | n/a | 2+2 | 24 months + 3 months | n/a | |
| **Lifshitz et al., 2012** | Microscope (n/a) | Perls’ blue | n/a | T1-weighted image enhancement due to gadolinium leakage | Contrast agent extravasates when BBB dysfunctional | Pre-post contrast MRI | 12% signal enhancement | n/a | Wild type mice | 5 | 16 months | n/a | 5 | 16 months | n/a | |
| **Klohs et al., 2013** | MRI (n/a) | SWI | Predominantly lobar | Median k-trans value within ROI (gadolinium leakage rate) | Contrast agent extravasates when BBB dysfunctional | DCE-MRI | n/a | n/a | Wild type mice | 15 | n.a | n/a | 15 | n/a | n/a | |
| **Winkler et al., 2001** | Microscope (n/a) | H&E, Perls’ blue | Neocortex, thalamus, and hippocampus | BBB leakage of HRP/Trypan blue | HRP/Trypan blue extravasate when BBB dysfunctional | Immunohistochemistry, confocal microscopy | n/a | n/a | Littermate control mice | 3 | 24 months | 0 | 3 | n/a | n/a | |

Abbreviation key: BBB = blood-brain barrier; sd = standard deviation; DCE-MRI = dynamic contrast enhanced magnetic resonance imaging; CE = contrast enhancement; ICH= intracerebral hemorrhage; CAA = cerebral amyloid angiopathy; APPsw, transgenic mouse model expressing the swedish mutation of the human amyloid precursor protein; DAB, 3, 3’H&E = hematoxylin and eosin; MMP= matrix metalloproteinase; CMB= cerebral microbleed; SP-SHR= stroke-prone spontaneously hypertensive rats; SWI = susceptibility weighted imaging; IgG= immunoglobulin G; HRP = horseradish peroxidase; ROI = region of interest ;

Table S4: methodology in human studies

| **Study** | **Hemorrhage marker( % study sample with hemorrhage)** | **Assessment method hemorrhage** | **Location hemorrhage** | **BBB dysfunction marker** | **Reason authors chose this marker** | **Assessment method BBB dysfunction** | **Mean(sd) marker of BBB dysfunction study sample** | **Mean(sd) marker of BBB dysfunction control sample** | **Control sample** | **Study sample** | | | **Control sample** | | | |
| --- | --- | --- | --- | --- | --- | --- | --- | --- | --- | --- | --- | --- | --- | --- | --- | --- |
|  |  |  |  |  |  |  |  |  |  | **size** | **age** | **% male** | **size** | **age** | | **% male** |
| **Plesea et al., 2005** | ICH symptoms/ pathologic evaluation (100%) | H&E | n/a | CD-34 immunoreactivity | Structural integrity ECs reflects BBB integrity | Immunohistochemistry | n/a | n/a | Absent | 82 | n/a | n/a | n/a | n/a | n/a | |
| **Hernandez-Guillamon et al., 2012 part 1** | MRI (100%) | T2*-weighted images | 100% lobar | MMP-2 and MMP-9 precursor concentrations in plasma | MMPs have been associated with BBB dysfunction | ELISA | MMP-2, 1459.29+/-330.58 ng/mL; MMP-9 77.95+/-53.56 ng/mL | MMP-2, 1458.99+/-355.25 ng/mL; MMP-9 66.54+/-38.10 ng/mL | Controls free of neurovascular and cardiovascular history | 33 | n/a | 61 | 21 | n/a | n/a | |
| **Hernandez-Guillamon et al., 2012 part 2** | ICH symptoms/pathologic evaluation (100%) | n/a | n/a | MMP-2 and MMP-9 immunoreactivity in cortical tissue | MMPs have been associated with BBB dysfunction | Immunoblotting/immunofluorescence | n/a | n/a | Controls that died without CAA and ICH | 4 | 85 | 25 | 3 | 76 | 66 | |
| **Hernandez-Guillamon et al., 2012 part 3** | ICH symptoms/pathologic evaluation (100%) | n/a | n/a | % of MMP-2 and MMP-9 positive vessels | MMPs have been associated with BBB dysfunction | immunohistochemistry | 20% within and 62% around CAA grade 2 vessels (n=60), 64% within and 100% around CAA grade 3 vessels (n=11), 50% within and 100% around CAA grade 4 vessels (n=24) | (10% within and 0% around CAA grade 0 vessels (n=30), 0% within and 27% around CAA grade 1 vessels (n=60); (20% within and 0% around control vessels (n=60) | Controls that died without CAA and ICH | 6 | 79 | 50 | 3 | 69 | 66 | |
| **Zhao et al., 2015** | Pathologic evaluation/microscope (n/a) | H&E, Perls’ blue | n/a | % of vessel immunopositive for MMP-9 | MMPs have been associated with BBB dysfunction | immunohistochemistry | 79%+/-23.9% of CAA affected vessels | 5% +/-1.3%of control vessels | Non-demented controls | 10 | 77 | 70 | 5 | 76 | 60 | |
| **Duits et al., 2015** | MRI (100%) | T2*-weighted images | 100% lobar | CSF levels of gelatinases and their tissue inhibitors | MMPs have been associated with BBB dysfunction | Beads-based immunoassay | n/a | n/a | AD/VaD without CMBs, cognitively normal controls | 34 | n/a | n/a | 27 + 26 | n/a | n/a | |
| **Cheng et al., 2014** | Microscope (n/a) | Perls’ blue | n/a | Occludin and ZO-1 expression in tissue and leptomeningeal vessels | Reduction of TJ proteins reflects BBB dysfunction | Immunohistochemistry/western blot | n/a | n/a | Controls that died without CAA | 9 | 89 | 22 | 10 | 87 | 50 | |
| **Hartz et al., 2012** | MRI (100%) | T2*-weighted images | 68% lobar ICH, 89% lobar CMB | % with CE lesions | Contrast agent extravasates when BBB dysfunctional | Post-contrast T1-weighted images | 2/19 (11%) cases | 17/19 (89%) cases | Absent | 19 | 75 | 53 | n/a | n/a | n/a | |
| **Van Assema et al., 2012** | MRI (100%) | SWI | 100% lobar | Binding potential of radiolabelled Pgp substrate (R)-[11C)verapamil |  | PET | 2.20+/-0.34 global; 2.15+/-0.42 frontal; 2.16+/-0.33 parietal; 2.31+/-0.35 temporal; 2.23+/-0.27 occipital; 2.17+/-0.44 posterior cingulate; 1.95+/-0.42 anterior cingulate; 3.12+/-0.52 medial temporal lobe; 1.94+/-0.28 cerebellum | 2.15+/-0.34 global; 2.10+/-0.33 frontal; 2.15+/-0.36 parietal; 2.23+/-0.39 temporal; 2.20+/-0.33 occipital; 2.12+/-0.44 posterior cingulate; 2.14+/-0.36 anterior cingulate; 2.82+/-0.48 medial temporal lobe; 2.03+/-0.27 cerebellum | AD without CMBs | 6 | 66 | 100 | 12 | 63 | 58 | |
| **Aksoy et al., 2013** | MRI (100%) | T2*-weighted images | 48% lobar, 28% deep, 16% infratentorial | Median k-trans value within ROI contralateral to ICH (gadolinium leakage rate) | Contrast agent extravasates when BBB dysfunctional | DCE-MRI | 12/25(48%) | n/a | Absent | 25 | 65 | 36 | n/a | n/a | n/a | |
| **Kidwell et al., 2011** | MRI (100%) | T2*-weighted images | n/a | Sulcal or ventricular CE | Contrast agent extravasates when BBB dysfunctional | FLAIR images | 39/46(85%) | n/a | Absent | 46 | 65 | 40 | n/a | n/a | n/a | |
| **McCourt et al., 2015** | CT (100%) | Non-contrast CT | 74% deep, 24% lobar, 2% infratentorial | Permeability surface area product in ipsilateral vs contralateral hemisphere | Contrast agent extravasates when BBB dysfunctional | Post-contrast CT | 4.2+/- 2.1 mL/100mL per minute | 3.7+/-1.6mL/100mL per minute | Absent | 53 | 71 | 72 | n/a | n/a | n/a | |
| **Xu et al., 2017** | ICH symptoms/CT (100%) | Non-contrast CT | 100% basal ganglia | Permeability surface area product in ipsilateral vs contralateral hemisphere | Contrast agent extravasates when BBB dysfunctional | Post-contrast CT | 1.18+/-0.50 mL/100g per minute | 1.18+/-0.56 mL/100g per minute | Absent | 54 | 58 | 65 | n/a | n/a | n/a | |
| **Tran et al., 2016** | ICH symptoms /microscope (100%) | H&E | n/a | Relative fluorescence units per gram tissue | Reduction of TJ proteins reflects BBB dysfunction | Fluorescence intensity analysis | n/a | n/a | Controls that died without ICH | 9 | 57 | 66 | 10 | 52 | 80 | |
| **Shams et al., 2016** | MRI (100%) | T2*-weighted imaging and SWI | n/a | CSF/serum albumin ratio | Elevated CSF/serum albumin ratio reflects BBB dysfunction | ELISA | 6.7 | 6.1 | Memory clinic patients without CMBs | 214 (118 multiple CMBs | n/a | n/a | 825 | n/a | n/a | |
| **Poliakova et al., 2016** | MRI (100%) | SWI | n/a | CSF/serum albumin ratio | Elevated CSF/serum albumin ratio reflects BBB dysfunction | Latex aggregation method and Brom-Cresol-Green method | 8.3+/-1.9 | 5.4+/-2.9 | Patients with cognitive decline without CMBs | 15 | n/a | n/a | 13 | n/a | n/a | |
| **Zhang et al., 2016** | MRI (100%) | SWI | 62% strictly lobar, | serum VEGF levels | VEGF plays an important role in the control of vascular permeability | ELISA | 336.72+/-15.18 pg/mL | 192.37+/-11.34pg/mL | AD without CMBs | 47 | 77 | 45 | 99 | 70 | 42 | |
| **Goos et al., 2012** | MRI (100%) | T2*-weighted images | 65% strictly lobar | CSF/serum albumin ratio | Elevated CSF/serum albumin ratio reflects BBB dysfunction | nephelometry | 6.7+/-3 | 7.4+/-4 | AD without CMBs | 26 | 67 | 62 | 26 | 67 | 62 | |

Abbreviation key: BBB = blood-brain barrier; sd = standard deviation; DCE-MRI = dynamic contrast enhanced magnetic resonance imaging; CE = contrast enhancement; ICH= intracerebral hemorrhage; ECs = endothelial cells; FLAIR = fluid-attenuated inversion recovery; ZO-1 = zona occludens 1; VaD = vascular dementia; AD = Alzheimer’s disease; CAA = cerebral amyloid angiopathy; VEGF = vascular endothelial growth factor; ELISA = enzyme linke immunosorbent assay; CSF = cerebrospinal fluid; pgp = p-glycoprotein; CT = computed tomography; H&E = hematoxylin and eosin; MMP= matrix metalloproteinase; CMB= cerebral microbleed; SP-SHR= stroke-prone spontaneously hypertensive rats; SWI = susceptibility weighted imaging; IgG= immunoglobulin G; HRP = horseradish peroxidase; ROI = region of interest

**References**

Herzog, R., Alvarez-Pasquin, M.J., Diaz, C., Del Barrio, J.L., Estrada, J.M., Gil, A., 2013. Are healthcare workers' intentions to vaccinate related to their knowledge, beliefs and attitudes? A systematic review. BMC public health 13, 154.

Sena, E., van der Worp, H.B., Howells, D., Macleod, M., 2007. How can we improve the pre-clinical development of drugs for stroke? Trends in neurosciences 30(9), 433-439.

Wells, G.A., Shea, B. O'Connell, D., Peterson, J., Welch, V., Losos, M., Tugwell, P., The Newcastle-Ottawa Scale (NOS) for assessing the quality of nonrandomized studies in meta-analyses.
